# Trajectories of growth and symptoms of attention-deficit/hyperactivity disorder in children: a longitudinal study

**DOI:** 10.1186/1471-2431-11-84

**Published:** 2011-10-10

**Authors:** Kati Heinonen, Katri Räikkönen, Anu-Katriina Pesonen, Sture Andersson, Eero Kajantie, Johan G Eriksson, Timo Vartia, Dieter Wolke, Aulikki Lano

**Affiliations:** 1 Institute of Behavioral Sciences, University of Helsinki, Helsinki, Finland; 2 Institute of Clinical Medicine, University of Helsinki, Helsinki, Finland; 3 Children's Hospital, Helsinki University Central Hospital and University of Helsinki, Helsinki, Finland; 4 National Institute for Health and Welfare, Helsinki, Finland; 5 Department of General Practice and Primary Health Care, University of Helsinki, Helsinki Finland; 6 Helsinki University Central Hospital, Unit of General Practice, Helsinki, Finland; 7 Folkhälsan Research Centre, Helsinki, Finland; 8 Children's and Adolescents Clinic Pikkujätti, Helsinki, Finland; 9 Department of Psychology and HSRI, University of Warwick, Coventry, UK

## Abstract

**Background:**

Empirical evidence suggests that prenatal growth is associated with attention deficit/hyperactivity disorder (ADHD) and its symptoms. Data on the importance of postnatal growth is, however scanty. We studied whether pre- and postnatal growth up to 56 months is associated with symptoms of ADHD in children.

**Method:**

A longitudinal regional birth cohort study comprising 893 children followed up to 56 months. The associations between pre- and postnatal growth and parent-rated ADHD symptoms of the child were analyzed with multiple linear regression analyses and repeated-measures analyzes of covariance.

**Results:**

Children born lighter, thinner, shorter, and with a smaller head circumference, adjusted for length of gestation, received higher parent-rated ADHD symptoms scores at 56 months. Further, smaller head circumference throughout the period of growth from birth up to 56 months was related to higher ADHD symptoms scores. The associations changed only little after adjusting for several pre- and neonatal factors. The associations were not modified by sex and there were no evidence of non-linear associations.

**Conclusions:**

Slower prenatal growth in weight, body-mass index, length, and head circumference may pose a risk for higher ADHD symptoms in childhood. The consistently smaller head circumference from birth up to 56 months characterizing children with higher ADHD symptoms may point to a lack of catch-up growth in head circumference in childhood as a predisposing factor.

## Background

Attention deficit/hyperactivity disorder (ADHD) is among the most common neuropsychiatric disorders in childhood with a worldwide prevalence rate of approximately 5% [1]. Behavioral symptoms characterizing ADHD are even more common, and include inattention, hyperactivity, and impulsiveness [2]. The etiology of ADHD is multifaceted. Together with genetic and environmental factors both pre- and postnatal growth may influence its development [3,4]. Indeed, mounting empirical evidence shows that prematurity and small body size at birth are associated with ADHD and its symptoms [5-11]. The evidence for the importance of postnatal growth is, however scanty and often confounded by stimulant medication used in treating ADHD symptomatology; stimulant-medication may *per se *compromise physical growth. Thus, it remains unknown if postnatal growth is etiologically associated with ADHD or is a consequence of the stimulant-medication [12]

In stimulant-naïve child and adolescent samples, those who gain higher scores in ADHD symptoms or who fulfill the criteria of the disorder have in cross-sectional studies been shown both to be shorter [13,14] or taller [15,16], to have higher weight [16] and both to have a lower [17] or a higher [16,18,19] body-mass index (BMI). Yet, to our knowledge only two studies to date have addressed the role of physical growth in ADHD: the first compared growth of 48 boys with ADHD in relation to population norms and found a distinct suppression of growth in height from 9 to 14 years, earlier onset of adolescent growth spurt and a higher velocity of growth in that moment [15]. The second study showed in 87 extremely-low-birth-weight boys and girls that growth in head circumference between birth and two years did not predict parent/teacher-rated ADHD at school-age [20]. The current study will test if growth in height, weight, BMI and head circumference from birth up to five, 20, and 56 months of age is associated with parent-rated ADHD symptoms at 56 months. Thus, the current study will add new information of the association between the postnatal growth from birth to preschool-age and symptoms of ADHD in a stimulant-naïve sample of girls and boys. By using different time points and a range of anthropometric measurements the study will also show whether there are particular periods of growth or measures that are specifically related to symptoms of ADHD.

## Methods

### Participants

The study cohort comprised infants participating in the Arvo Ylppö Longitudinal Study (AYLS) [21,22]. Of a total of 15,311 deliveries in the seven maternity hospitals in the county of Uusimaa, Finland, we identified all live-born infants between March 15, 1985 and March 14, 1986 who were admitted to the neonatal wards of the obstetric units or transferred to the Neonatal Intensive Care Unit of the Hospital for Children and Adolescents, University of Helsinki, within 10 days of birth. The population ranged from severely ill preterm infants to infants born at term requiring only brief inpatient observation. In total, there were 1,535 admitted infants (n = 867 boys). In addition, 658 infants (n = 328 boys), born during the same period but not admitted to the neonatal ward, were randomly recruited from the three largest maternity hospitals in the study area. Participants were invited to clinical follow-ups at 5 and 20 months of corrected age and at 56 months of age. The study protocol was approved by the Ethical Committees of Helsinki City Maternity Hospital, Helsinki University Central Hospital, and Jorvi Hospital, and the parent(s) gave their informed consent.

Of the 2,193 infants 2,125 survived to 56 months of age. We excluded 80 infants with congenital malformations and chromosomal abnormalities, and Mendelian disorders potentially affecting growth. At least one birth size measure, control variable and/or parental ratings of ADHD at 56 months was lacking for 1,232 children. Thus, the participants in the present study included 893 children: boys (n = 490) and girls (n = 403). The original cohort is described in detail elsewhere [22,23].

The study participants (n = 893) did not differ from those who were lost to follow-up due to lack of information on any birth size measure, gestational age and/or pre- and neonatal control variables (n = 225) in sex, multiple pregnancy, mother's height, on BMI at five months, or in any body size measures at 20 and 56 months (all *p*-values > .05). However, those lost to follow-up were more likely to have been admitted to a neonatal ward, to come from less educated families, to have younger mothers, and mothers who smoked more during pregnancy. Furthermore, they were lighter, shorter and had smaller head circumference at birth, and at 5 months, and were also thinner at birth, born earlier and less likely to be breast fed (all *p*-values < .04). Further, the study participants (n = 893) did not differ from those who were lost to follow-up due to lack of information on parental rated ADHD symptoms (n = 927) in any body size or control variables, except that those who did not had ADHD symptoms scores available were shorter at 5 and 20 months and their mothers had smoked more during pregnancy (*p*-values < .049). As the ADHD symptoms were measured at the age of 56 months the participants were not yet treated with stimulant medication, even if symptoms of ADHD might already be present.

### Measures

#### Body size at birth, and in infancy and childhood

Data on the newborns' date of birth, weight (g), length (cm) and head circumference (cm) were extracted from hospital birth records, and BMI was calculated (kg/m^2^). The same anthropometric measures were taken during the clinical visits at five and 20 months of corrected age and at 56 months.

#### Growth

The growth variables were the standardized residuals from the linear regression models where body size at each time point was regressed on corresponding size measures at all earlier time points, thus creating completely uncorrelated residuals reflecting growth conditional on previous history. The residuals provided information on whether growth by a particular age was faster or slower than would have been predicted from previous measurements.

#### Gestational age

Gestational age was determined from the date of the mother's last menstrual period and/or ultrasound examination. All the infants were given an assessment of clinical maturity [24]. When there was a difference in the estimates of more than two weeks, gestational age was corrected according to Dubowitz maturity scale, except for preterms under 32 weeks of gestational age.

#### Parental Ratings of ADHD symptoms

At the 56-month follow-up the parents evaluated the child's ADHD symptoms with the Conners' Hyperactivity Index -parent version [25]. This index is composed of ten items rated on a four-point scale (0 = not at all to 3 = very much). Sample items are: "Inattentive, easily distracted" and "Restless or overactive". The items were summed with higher scores reflecting higher levels of ADHD symptoms. Cronbach's alpha was .85.

#### Potential covariates

We tested if the associations between body size/growth variables and ADHD symptoms were confounded by the child's sex, gestational age, admission to neonatal ward during the first ten days, multiple pregnancy (singleton vs. multiple), mother's smoking during pregnancy (none, 1-10, > 10 cigarettes/day), parental education (four point scale of highest self-reported level of education of either parent: from high = university education to low = elementary school education or less), maternal age (years), maternal height (cm).

### Statistical analysis

Body size at birth and at five, 20, and 56 months of age was converted into z scores (Mean = 0; Standard Deviation (*SD*) = 1) by sex, and birth size measures additionally by gestational age. The standardized scores represent the difference from mean value for the sample of control children participating in the current study (i.e. those who were not admitted to neonatal ward).

As the primary data analytical tool, we used multiple linear regression analysis. We examined, first, if body size at birth, and at five, 20 and 56 months, and, second, if growth from birth to five, 20 and 56 months of age, conditional on previous history, predicted the child's ADHD symptoms at 56 months as a continuous score. As the associations are not necessarily linear, we also tested the potential non-linearity of the associations by entering squared terms of body size at each time point into the regression equation following the linear terms.

Finally, we examined trajectories of growth by using repeated-measures analysis of covariance (ANCOVA). In the ANCOVA body size at birth, five, 20 and 56 months of age represented the within-person dependent variables, and quintiles of ADHD symptoms at 56 months represented the independent between-person grouping variable. All associations were tested after controlling for the sex of the child, and then further controlling for pre- and neonatal covariates.

We also re-ran analyzes in a subgroup of children not admitted to neonatal ward during first 10 days, and who had birth weight ≥ 2,500 g and were born at term (≥ 37 weeks of gestation). This remaining subgroup included 293 children.

## Results

Table 1 shows characteristics of the sample divided by sex. Compared with girls, boys had higher scores on ADHD-symptoms at 56 months. Boys were bigger at all measurement points and were more likely to have been admitted to a neonatal ward. In addition, we tested interactional effects of sex by adding an interaction term 'sex × body size (each anthropometric variable at each time point in separate models)' into the regression equation in addition to the main effects of these two centered variables in predicting ADHD-symptoms. These analyses revealed no significant interactions (all *p*-values > .13). All the main results are presented in the pooled sample. When testing for associations between covariate variables and ADHD-symptoms, we found that after controlling for the child's sex, children of younger mothers, and of parents with a lower educational level had higher ADHD-symptoms scores (*p*-values < .002; all other *p-values *> .13).

**Table 1:** Characteristics of the sample by sex

		Boys			Girls		
	n	Mean (SD)/n (%)		n	Mean (SD)/n (%)		
Parental rated ADHD-symptoms	490	9.5	5.0	403	8.4	4.7	*
*Body size*							
At birth							
Weight, kg	490	3.4	0.8	403	3.3	0.7	*
Length, cm	490	49.6	3.6	403	48.7	3.2	*
Head circumference, cm	490	35.0	2.2	403	34.3	2.1	*
BMI, kg/m^2^	490	13.5	1.8	403	13.5	1.8	
At 5 months							
Weight, kg	482	7.6	0.9	394	7.0	0.9	*
Length, cm	478	66.5	2.3	388	64.5	2.2	*
Head circumference, cm	480	43.6	1.2	391	42.3	1.1	*
BMI, kg/m^2^	476	17.2	1.5	388	16.8	1.5	*
At 20 months							
Weight, kg	457	12.1	1.4	376	11.4	1.4	*
Height, cm	444	84.6	3.1	369	83.2	3.2	*
Head circumference, cm	459	49.4	1.4	381	48.1	1.2	*
BMI, kg/m^2^	440	16.8	1.4	369	16.4	1.3	*
At 56 months							
Weight, kg	482	18.5	2.6	393	17.8	2.7	*
Height, cm	470	108.6	4.4	390	107.3	4.6	*
Head circumference, cm	476	52.3	1.5	392	51.0	1.3	*
BMI, kg/m^2^	469	15.6	1.5	387	15.4	1.5	*
*Covariates*							
Gestational age, weeks	490	38.4	2.8	403	38.7	2.6	
Admission to neonatal ward (n, %)	490	341	69.6	403	251	62.3	*
Multiple pregnancy (n, %)	490	21	4.3	403	21	5.2	
Maternal smoking during pregnancy (n, %)	490			403			
> 10 cigarettes/day		27	5.5		25	6.2	
1-10 cigarettes/day		82	16.7		71	17.6	
None		381	77.8		307	76.2	
Mother's							
Height, cm	490	164.4	5.4	403	164.3	5.5	
Age, Years	490	29.4	5.1	403	29.4	5.0	
Parental education (n, %)	490			403			
I		58	11.8		48	11.9	
II		171	34.9		154	38.2	
III		151	30.8		107	26.6	
IV		110	22.4		94	23.3	
Breast fed (yes: n, %)	490	457	93.3	403	378	93.8	

Note. ADHD = Attention-Deficit/Hyperactivity Disorder, BMI = Body-mass index, Parental education I = Low to IV = High, * P < 0.05.

### Relative body size and parent-rated ADHD symptoms at 56 months

Table 2 shows that lower weight, shorter length, smaller head circumference and lower BMI at birth were significantly associated with higher ADHD symptoms scores after controlling for child's sex. Table 2 also shows that smaller head circumference at five, 20 and 56 months of age were significantly associated with higher ADHD symptoms scores. After controlling for pre- and neonatal covariates, the association between head circumference at 20 months and ADHD symptoms were rendered to marginal significance (from *p *= .02 to *p *= .08), whereas the previously marginally significant association between lower BMI at 56 months and higher ADHD symptoms became significant (from *p *= .09 to *p *= .04).

**Table 2:** Relative body size at birth, and up to 56 months of age and ADHD-symptoms

	Model I			Model II		
		
	Effect size in SD units	(95% CI)	P-value	Effect size in SD units	(95% CI)	P-value
At birth						
Weight	-0.39	(-0.65 to -0.13)	0.003	-0.41	(-0.69 to -0.14)	0.003
Length	-0.32	(-0.58 to -0.06)	0.02	-0.33	(-0.61 to -0.05)	0.02
Head circumference	-0.40	(-0.68 to -0.12)	0.01	-0.36	(-0.65 to -0.07)	0.01
BMI	-0.33	(-0.59 to -0.07)	0.01	-0.33	(-0.60 to -0.06)	0.02
At 5 months						
Weight	-0.16	(-0.45 to 0.13)	0.28	-0.19	(-0.53 to 0.15)	0.27
Length	-0.12	(-0.42 to 0.18)	0.44	-0.1	(0.46 to 0.26)	0.57
Head circumference	-0.39	(-0.69 to -0.10)	0.01	-0.31	(-0.61 to -0.01)	0.04
BMI	-0.13	(-0.43 to 0.16)	0.37	-0.17	(-0.50 to 0.15)	0.29
At 20 months						
Weight	-0.11	(-0.43 to 0.21)	0.49	-0.13	(-0.46 to 0.20)	0.44
Length	-0.05	(-0.36 to 0.26)	0.73	-0.02	(-0.35 to 0.31)	0.90
Head circumference	-0.36	(-0.68 to -0.05)	0.02	-0.29	(-0.61 to 0.04)	0.08
BMI	-0.14	(-0.46 to 0.19)	0.42	-0.19	(-0.52 to 0.14)	0.26
At 56 months						
Weight	-0.04	(-0.36 to 0.25)	0.72	-0.1	(-0.43 to 0.24)	0.57
Length	0.09	(-0.22 to 0.40)	0.56	0.15	(-0.20 to 0.51)	0.40
Head circumference	-0.40	(-0.71 to -0.10)	0.01	-0.34	(-0.65 to -0.03)	0.03
BMI	-0.27	(-0.57 to 0.04)	0.09	-0.34	(-0.67 to -0.01)	0.04

ADHD = Attention-Deficit/Hyperactivity Disorder, BMI = Body-mass index

Model I: After controlling for child's sex; Model II: after controlling for child's sex, gestational age, admission to neonatal ward, multiple pregnancy, mother's smoking during pregnancy, parental education, maternal age, and maternal height.

### Growth and parent-rated ADHD symptoms at 56 months

#### Growth conditional on history

After controlling for child's sex there were no significant associations between growth (conditional on history) and ADHD symptoms. However, after controlling for the rest of the covariates the previously marginally significant association (*p *= 0.06) between a slower gain in BMI from 20 to 56 months and higher ADHD symptoms scores became significant (B = -0.37, 95% Confidence Interval (*CI*): -0.70 to -0.32, *p *= .03) (Table 3.).

**Table 3:** Associations between growth (conditional on history) and parental rated symptoms of Attention deficit/hyperactivity disorder (ADHD)

Growth variable	Parental rated symptoms of ADHD, Unstandardized regression coefficient (95% CI)	
	**Model I**	**Model II**

Weight		
At birth	-0.37 (-0.64 to -0.09)*	-0.37 (-0.66 to -0.08)*
Growth from birth to 5 months	0.02 (-0.33 to 0.37)	-0.01 (-0.36 to 0.34)
Growth from 5 to 20 months	0.11 (-0.22 to 0.43)	0.09 (-0.24 to 0.41)
Growth from 20 to 56 months	-0.03 (-0.36 to 0.30)	-0.09 (-0.42 to 0.24)
Length/height		
At birth	-0.29 (-0.56 to -0.01)*	-0.28 (-0.57 to 0.02)
Growth from birth to 5 months	0.12 (-0.24 to 0.49)	0.12 (-0.25 to 0.48)
Growth from 5 to 20 months	0.03 (-0.29 to 0.36)	0.06 (-0.27 to 0.39)
Growth from 20 to 56 months	0.11 (-0.24 to 0.45)	0.07 (-0.27 to 0.42)
Head circumference		
At birth	-0.37 (-0.67 to -0.07)*	-0.33 (-0.63 to -0.02)*
Growth from birth to 5 months	-0.18 (-0.52 to 0.16)	-0.15 (-0.49 to 0.20)
Growth from 5 to 20 months	-0.23 (-0.58 to 0.12)	-0.19 (-0.54 to 0.16)
Growth from 20 to 56 months	-0.25 (-0.59 to 0.10)	-0.25 (-0.59 to 0.09)
Body-mass-index		
At birth	-0.22 (-0.50 to 0.05)	-0.22 (0.51 to 0.07)*
Growth from birth to 5 months	-0.02 (-0.36 to 0.32)	-0.08 (-0.41 to 0.27)
Growth from 5 to 20 months	0.07 (-0.27 to 0.41)	0.03 (-0.31 to 0.37)
Growth from 20 to 56 months	-0.31 (-0.65 to 0.02)	-0.37 (-0.70 to -0.03)*

Birth size, growth rates from birth to 5 months, from 5 to 20 months, and from 20 to 56 months are considered simultaneously. The growth variables are expressed as standardized residuals of regression of size at each age stage on size at previous age stage(s), that is, growth conditional on history. Model I includes adjustments for child's sex; Model II includes adjustments for child's sex, gestational age, admission to neonatal ward during the first ten days, multiple pregnancy, mother's smoking during pregnancy, parental education, maternal age and maternal height. * P < 0.05

#### Growth trajectories

**Figure 1:**
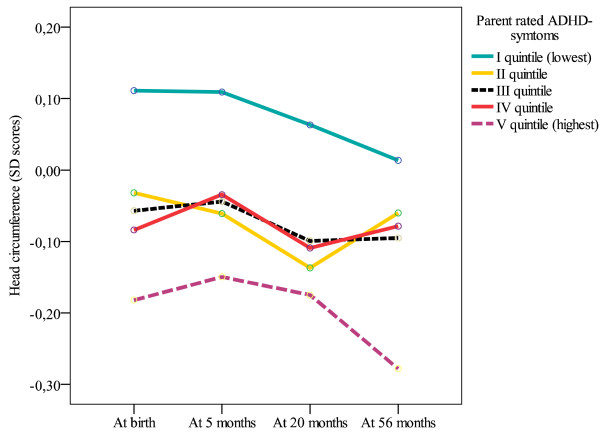
**Head circumference up to 56 months of age and Attention-Deficit/Hyperactivity Disorder (ADHD) symptoms**. The associations between head circumference (Standard Deviation (SD) scores) at birth, and at five, 20 and 56 months age and parent rated ADHD symptoms as quintiles after controlling for child's sex, gestational age, admission to neonatal ward, multiple pregnancy, mother's smoking during pregnancy, parental education, maternal age, and maternal height.

Figure 1 displays the growth trajectory of head circumference in quintiles of ADHD symptoms scores in a fully controlled model. The pattern of the growth trajectory across time was not significantly different between the ADHD symptoms group (ADHD grouping × time, *p *= .93). However, children who had ADHD symptoms scores in the highest quintile displayed the lowest head circumference and children having the lowest ADHD symptoms scores displayed the highest head circumference from birth to 56 months (Mean difference in *SD *units = 0.27, 95% *CI *= 0.06 to 0.49, *p *= .01). Head circumferences of the children with ADHD symptoms scores in the middle three quintiles fell between these two extreme quintiles. The analyses of other growth measures by quintiles of ADHD symptoms were not statistically significant.

### Associations in a subgroup of full term and normal birth weight children not admitted to neonatal ward

Finally, we tested whether the observed results remained similar in a subgroup of children who were not admitted to the neonatal ward, had birth weight ≥ 2,500 g and were born at term (≥ 37 weeks of gestation) (n = 293). We found that in a fully controlled model lower weight (*p *= 0.005), smaller head circumference (*p *= 0.06) and lower BMI (*p *= 0.001) at birth were significantly or marginally significantly associated with higher ADHD symptoms scores. However, birth length was not associated with the ADHD symptoms (*p *= 0.27). Further, smaller head circumference at five (*p *= 0.05), 20 (*p *= 0.04) and 56 months of age (*p *= 0.03) were associated with higher ADHD symptoms scores. None of the previously found associations between relative BMI at 56 months and growth in BMI from 20 to 56 months and ADHD symptoms were present in this subgroup. Finally, the growth trajectory of head circumference in quintiles of ADHD symptoms scores was similar to that found in a total sample. Children who had ADHD symptoms scores in the highest quintile displayed the lowest head circumference and children having the lowest ADHD symptoms scores displayed the highest head circumference from birth to 56 months (Mean difference in *SD *units = 0.37, 95% *CI *= 0.02 to 0.72, *p *= .04).

## Discussion

We found in a sample of 893 Finnish children that those born lighter in weight, thinner in BMI, shorter in stature and smaller in head circumference were rated by their parents as having higher ADHD symptoms scores at 56 months. Further, we found that a smaller head circumference throughout the period of growth from birth to 56 months was related to higher ADHD symptoms. We also found that a slower gain in BMI from 20 to 56 months and a lower relative BMI at 56 months was associated with higher ADHD symptoms. Controlling for several pre- and neonatal covariates had little effect on the results. The associations were not modified by sex and there were no non-linear associations.

We also found a similar pattern of findings in a subgroup of term (≥ 37 weeks of gestation), normal birth weight (≥ 2,500 g) children not admitted to neonatal ward (n = 293) with three clear exceptions: the association between birth length, growth in BMI from 20 to 56 months and the relative BMI at 56 months and ADHD symptoms scores were turned to non-significant.

There are several strengths in this study. We were able to examine the early childhood growth using multiple anthropometric variables in a prospective study design. Most of the existing studies have been conducted with data derived from clinical practice, which may favor a higher than expected rate of positive findings [26]. Also studies with stimulant-naïve sample are needed to evaluate whether potential growth deficits may indeed be etiologically related to ADHD and not a consequence of stimulant-medication used to treat symptomatology [12]. Further, we had the whole birth weight range available and could control for several variables that may have an impact on growth and/or ADHD symptoms. Controlling for covariates increases the likelihood that the findings are true and not due any confounding factor related to the both independent and dependent variables. We could also exclude children with severe conditions that could have potentially confounded the findings.

There are also some limitations. For practical reasons, several parents did not receive or fill in the ADHD questionnaire. However, those who did and did not receive/fill in the questionnaire did not differ from each other in most of the measured variables. However, compared to those lost to follow-up the participants were taller at five and at 20 months, and their mothers smoked less during pregnancy. Therefore, the results may be more characteristic of children exposed to less adverse pre- and postnatal environments. Further, we had no information on the parental ADHD symptoms and were, thus, unable to take potential genetic susceptibility into account. Neither, did we have information on environmental postnatal factors as non-optimal parenting [27,28] and family adversity [29] that have been shown to be related to symptoms of ADHD. However, we have controlled for parental education, which is a crude proxy of several environmental adversities. As we have used multiple measures of body size at four different time points, we have ended up making multiple tests. However, we decided not to use the correction for multiple testing (e.g., Bonferroni correction). First, as the number of Type I errors cannot decrease without increasing type II errors [30]. Second, theoretical assumption behind the correction of multiple testing is that all null hypotheses are true simultaneously, which was not of interest of our study. It is suggested simply describing carefully the used analyses is usually the most rational way of dealing with multiple testing [31].

We have previously shown with the same data that small for gestational age (SGA) status, rather than prematurity *per se*, may increase risk for symptoms of ADHD in young children [9]. While current findings showing associations between smaller body size in weight, length, head circumference and BMI at birth and higher ADHD symptoms scores are in accordance with our and majority of others previous findings [5-8,10,11], they also extend them in significant ways. Of the few previous studies focusing on children born across the whole gestational age/birth weight range [e.g., [9,32]] to our knowledge none has focused on birth weight, length, head circumference and BMI in a same study. In our cohort, birth weight and head circumference seem to have the strongest associations with parent rated ADHD symptoms.

With regard to postnatal growth, to our knowledge there are only three prior studies focusing on postnatal head circumference and ADHD or its symptoms. Ptcak et al. [33] suggested that 4-16 years-old boys with diagnosed ADHD have a smaller head circumference than the normal non-clinical population, although the association was only marginally significant among those boys who did not receive medication (n = 52, *p *= .07). Hack et al. [34] found among 249 very-low-birth-weight children that those who had a subnormal head size (less than the mean -2 SD for age) at 8 months of corrected age had a higher incidence of hyperactivity at 8-9 years. This association became, however, non-significant after controlling pre- and neonatal covariates. Finally, Stathis et al. [20] failed to find any significant associations among 87 extremely-low-birth weight born children between subnormal head circumference (comparison to norm z-scores) or head growth velocity from birth to 2 years and parent/teacher rated ADHD diagnoses at school-age. Because of differences in age of the samples and periods of growth, direct comparison of our results showing an association between a smaller head circumference throughout the period of growth from birth to 56 months with higher levels of ADHD symptoms at 56 months, with earlier findings is not possible. The current study is the only one that presents results in a relatively large cohort of children born across the whole birth weight range.

The mechanisms explaining the association between postnatal head circumference and ADHD symptoms remain speculative. Head circumference has been shown to correlate highly with total brain volume (from 1.7 to six years r = .93) [35]. Smaller total brain volume, in turn, has been shown to be related to ADHD in several studies [36]. Further, our results showing that those having the highest levels of parent rated ADHD symptoms were those who had the smaller head circumferences already at birth and had a persistently smallest head circumference across the early childhood pointing, at least partly, to the compromised prenatal period the effects of which the postnatal growth has not been able to correct. The longitudinal growth curves of total brain volumes have been shown to be roughly parallel, although at different levels, between patients with ADHD and healthy controls [37] suggesting that developmental processes are essentially healthy in ADHD, and that ADHD symptoms appear to reflect fixed earlier neurobiological insults or abnormalities. Another study [38] provided evidence that the pattern of brain maturation in ADHD was delayed, rather than abnormal. As the brain volume usually reaches its maximum volume by early adolescence [39] the age range of the participants in the current study does not allow conclusions whether the association between smaller head circumference and higher levels of ADHD symptoms reflect slow maturation that will disappear later.

We also found that lower relative BMI at 56 months and slower gain in BMI from 20 to 56 months was related to higher ADHD symptoms. Earlier studies have shown that ADHD or its symptoms are associated with higher BMI [16,18,19] rather than with thinness [17], or have not found associations with BMI [14,40,41]. All previous studies have, however, studied older participants or used diagnoses of ADHD. It should also be noted that our results became non-significant after excluding the participants who were admitted to neonatal ward during first ten days, were born preterm or had a birth weight < 2,500 g suggesting the stronger relationship among those born with compromised prenatal period or early health problems. However, our findings of slower gain in BMI may also reflect the measurement period from 20 to 56 months. At that time children start to move more by themselves. They may burn a lot of energy while hyperactive, but the ADHD-related abnormal eating behaviors (e.g. binge eating) that are present in older individuals may only have a minor role on the choices and amount of food consumed ^see ^[42].

Earlier studies on height and ADHD have been mainly cross-sectional and inconsistent in their findings [13-16,43]. The one existing longitudinal study focusing on growth in height among 48 ADHD diagnosed boys from two to 17 years of age found a distinct suppression of growth in height from nine to 14 years, earlier onset of adolescent growth spurt and a higher velocity of growth in that moment [15]. That study however differed from the current study in measurement of ADHD and in age range. Our longitudinal findings that parental rated ADHD symptoms at 56-months are not related to relative height at successive time points or to growth trajectories during the first years extend the knowledge of prior studies to years before ADHD-diagnoses are usually made.

## Conclusions

In summary, the results point to the importance of a slower prenatal growth. The consistently smaller head circumference from birth to 56 months characterizing children with higher ADHD symptoms scores may point to the importance of a lack of catch-up growth in head in childhood as another predisposing factor. However, it should be kept in mind that the associations we found were relatively modest in effect size. Measurements of body size are nevertheless crude measurements of environmental conditions early in life, and any association may give a hint of a biologically significant relationship. Further, studies are clearly needed to illuminate the association between growth and ADHD throughout the entire period of growth.

## Competing interests

The authors declare that they have no competing interests.

## Authors' contributions

KH and KR made substantial contributions to analysis and interpretation of data and have been involved in drafting the manuscript and revising it critically for important intellectual content. A-KP, SA, EK, JGE have contributed to analysis and interpretation of data, and have been involved in revising manuscript critically for important intellectual content; DW has made substantial contributions to conception and design, and to interpretation of data, and has revised manuscript critically for important intellectual content. TV, AL have made substantial contributions acquisition of data and have been involved in revising manuscript critically for important intellectual content. All authors read and approved the final manuscript.

## Pre-publication history

The pre-publication history for this paper can be accessed here:

http://www.biomedcentral.com/1471-2431/11/84/prepub
